# Drug repurposing—a promising approach for patients with angina but non-obstructive coronary artery disease (ANOCA)

**DOI:** 10.3389/fcvm.2023.1156456

**Published:** 2023-06-16

**Authors:** Johanna McChord, Valeria Martínez Pereyra, Sarah Froebel, Raffi Bekeredjian, Matthias Schwab, Peter Ong

**Affiliations:** ^1^ Department of Cardiology and Angiology, Robert-Bosch-Krankenhaus, Stuttgart, Germany; ^2^ Dr. Margarete Fischer-Bosch Institute of Clinical Pharmacology, Stuttgart, Germany; ^3^ Departments of Clinical Pharmacology, and Biochemistry and Pharmacy, University Tübingen, Tübingen, Germany

**Keywords:** drug repurposing, sGC stimulators, ET-1 receptor blockers, refractory angina, ANOCA

## Abstract

In today’s era of individualized precision medicine drug repurposing represents a promising approach to offer patients fast access to novel treatments. Apart from drug repurposing in cancer treatments, cardiovascular pharmacology is another attractive field for this approach. Patients with angina pectoris without obstructive coronary artery disease (ANOCA) report refractory angina despite standard medications in up to 40% of cases. Drug repurposing also appears to be an auspicious option for this indication. From a pathophysiological point of view ANOCA patients frequently suffer from vasomotor disorders such as coronary spasm and/or impaired microvascular vasodilatation. Consequently, we carefully screened the literature and identified two potential therapeutic targets: the blockade of the endothelin-1 (ET-1) receptor and the stimulation of soluble guanylate cyclase (sGC). Genetically increased endothelin expression results in elevated levels of ET-1, justifying ET-1 receptor blockers as drug candidates to treat coronary spasm. sGC stimulators may be beneficial as they stimulate the NO-sGC-cGMP pathway leading to GMP-mediated vasodilatation.

## Introduction

The concept of drug repurposing (also called drug repositioning, reprofiling or re-tasking) refers to the identification of a new indication for an already existing and authorised drug. It is a way of making new treatment options available to patients. The overall costs for a new drug to appear on the market can exceed 1 billion US$ and many drugs fail at clinical trials or the approval stage (1). Since approved drugs have already undergone the path of development and are well characterized in terms of targets, efficacy and side effects, using an existing drug for a novel indication is a promising concept.

Drug repurposing already plays a significant role, particularly in oncology and the COVID-19 pandemic response. There is in fact a frequent coexistence of cancer and cardiovascular conditions. Clinical and preclinical data propose their interdependence because of their aging-related nature and pathophysiological associations (2). Cancer is acknowledged as an evolutionary adaptive form proficient enough to take advantage of the body’s cardiovascular-regulating mechanisms, such as RAS, adrenergic system, coagulation system, and sodium/potassium (Na^+^ /K^+^)-ATPase pump system, in order to secure its own survival. In accordance with this hypothesis, numerous cardiovascular system-regulating drugs show anticancer activity, which makes them potential candidates in oncological adjuvant therapy (3). Remarkably, the idea of drug repurposing from non-cancer indications to novel applications in oncology has led aspirin into the prophylaxis of colorectal cancer and thalidomide into the treatment of multiple myeloma (4).

Repurposed drugs for COVID-19 therapy have the great advantage of fast applicability in clinical practice, since saving time is crucial for pandemic control. A comprehensive list of repurposed and potentially repurposed drugs for COVID-19 is given by Sanders et al. (5) and Ngan et al. (6).

This review focuses on the advantage of drug repurposing, the different approaches to this concept as well as successful and promising examples in the context of cardiology.

## Drugs made new in cardiology

Since mortality of cardiovascular diseases remains high in industrial countries despite a large number of available drugs, major research on pharmacological treatment is needed (7). There have been numerous examples of drug repurposing of cardiovascular drugs (3) such as anti-inflammatory agents (8), interleukin receptor antagonists (Canakinumab-CANTOS) (9) and IL-6 alpha antibodies, which were initially prescribed for rheumatoid arthritis and are currently being investigated for the treatment of endothelial dysfunction and atherosclerotic disease (10).

Although the pathogenesis of cardiovascular disease is complex, contributing factors have been thoroughly investigated. For instance, patients with other but cardiac diseases, e.g., rheumatoid arthritis have been described to exhibit higher infarction rates (11). The intertwining of different diseases and the effect they have on the cardiovascular system is apparent. It is conceivable that systemic diseases, such as chronic inflammatory diseases or cancer affect the cardiovascular system, e.g., by alterations of the endothelium or cardiovascular-regulating mechanisms, which makes drug repurposing in cardiology even more promising. Table 1 lists a small number of selected cardiovascular drugs which have been repurposed for new indications. It also indicates the phases of clinical trials in which some of these drugs are currently being tested for new indications.

**Table 1:** Selected examples of drug repurposing in cardiology.

Drug	Old indications	New indications
Acetylsalicyclic acid	Pain, Fever, Inflammation	Coronary artery disease, Secondary prophylaxis after stroke or myocardial infarction, Prevention of pre-eclampsia
Carvedilol	Hypertension	Angina pectoris, Heart failure Discontinued: Arrhythmias, Ischemic heart disease, Myocardial infarction, Post-traumatic stress disorders, Reperfusion injury
Clonidine	Hypertension	Glaucoma, Attention deficit hyperactivity disorder (ADHD), Alcohol withdrawal syndrome Phase III: Sciatica Discontinued: Stomatitis
Colchicine	Gout, Familial mediterranean fever	Coronary artery disease Phase III: Myocardial infarction ineffective: COVID-19 pneumonia
Empagliflozin	Type 2 diabetes mellitus	Marketed: Chronic heart failure Preregistration: Renal failure Phase III: Acute heart failure, Gestational diabetes, Insulin resistance, Type 1 diabetes mellitus Phase II: Decompensated heart failure
Minoxidil	Hypertension	Androgenic alopecia
Rivaroxaban	Deep vein thrombosis, Embolism, Pulmonary embolism, Stroke, Thromboembolism, Thrombosis, Venous thromboembolism	Registered: Acute coronary syndromes, Cardiovascular disorders Phase III: Arterial thrombosis, Peripheral arterial disorders Phase II: Coronary thrombosis Phase I: Antiphospholipid syndrome
Sildenafil	Pulmonary arterial hypertension	Erectile dysfuntion Phase III: Diastolic heart failure, Heart failure Discontinued: Angina pectoris, Eye disorders, Female sexual dysfunction, Meniere’s disease

The information of Table 1 were taken from the database on https://adisinsight.springer.com/ and represent the status as of 05/24/2023.

### Drug repurposing for treatment of ANOCA patients

**Figure 1:**
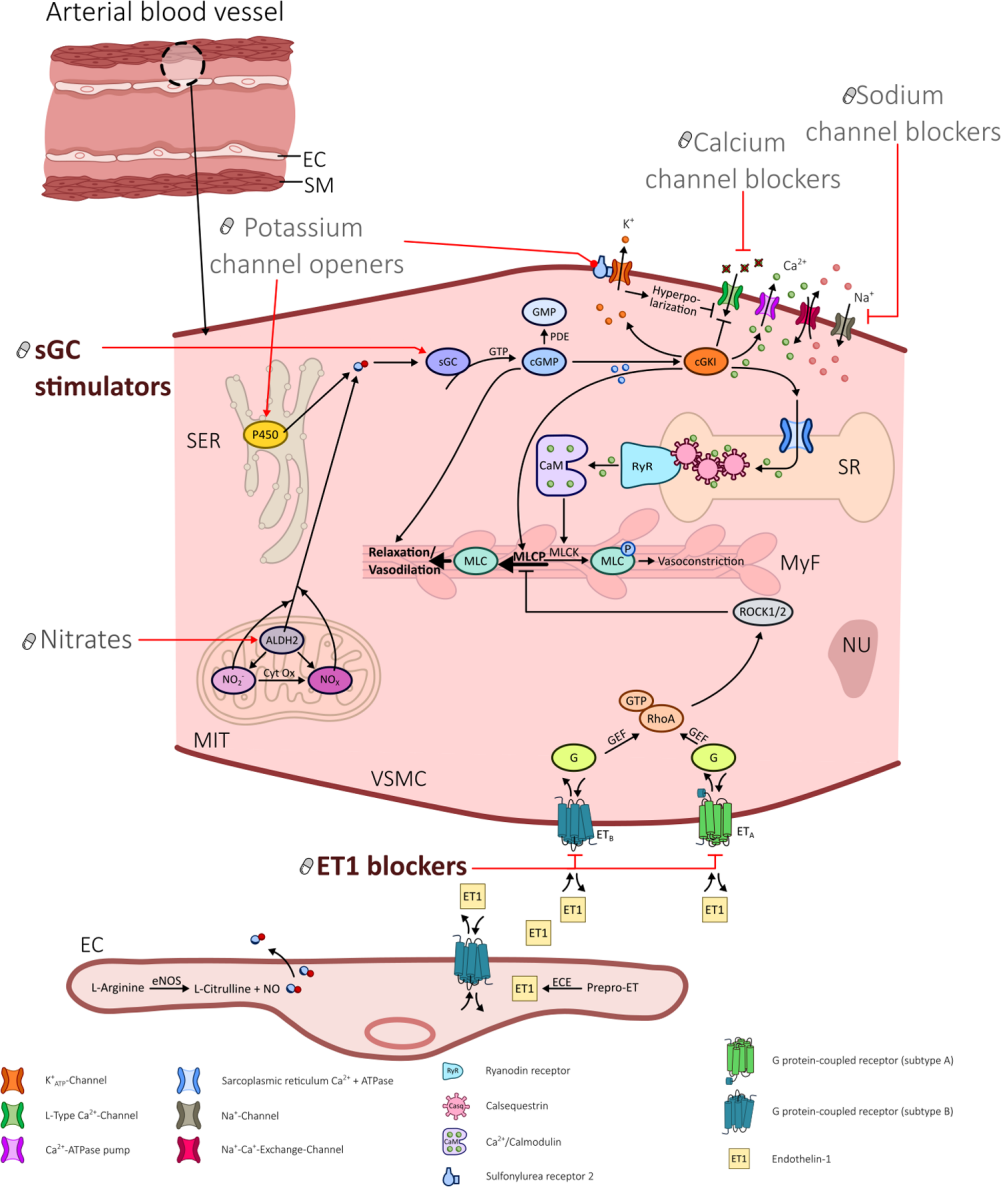
Illustration portraying the various influences of different drugs in altering signaling pathways in vascular smooth muscle cells in coronary arteries.

In patients with symptoms suggestive of obstructive coronary artery disease who undergo diagnostic coronary angiography about 40% have entirely normal arteries or non-obstructive coronary artery disease (12–14). This heterogeneous group of ANOCA patients frequently suffers from coronary vasomotor disorders such as coronary spasm and/or impaired microvascular vasodilatation and shows a high rate of non-responders to standard therapy (15–17). Unfortunately, there are no therapy guidelines for ANOCA yet because large-scale evidence-based data are still lacking. In 2020 an EAPCI Expert Consensus Document on ischemia with non-obstructive coronary arteries endorsed by the “Coronary Vasomotor Disorders International Study Group” (COVADIS) was published which describes therapy recommendations (18). In addition, the one year outcomes of angina management guided by invasive coronary function testing (CorMicA) (19) highlight that a stratified medical therapy based on an invasively diagnosed endotype of coronary vasomotion disoders significantly improves angina severity, quality of life, illness perception, and global treatment satisfaction. The investigators of the CorMicA trial used common antianginal medications (such as beta-blockers, calcium channel blockers, nicorandil, ranolazine, and nitrates).Those drugs interfere in the signaling pathways of vascular smooth muscle cells in coronary arteries and result in vasodilation, as illustrated in Figure 1:

1. Calcium channel blockers (CCB) inhibit L-type Ca^2+^ channels and reduce intracellular Ca^2+^.
2. Nitrates activate mitochondrial aldehyde dehydrogenase and increase intracellular NO availability.
3. Potassium channel openers (PCO) such as nicorandil bind to and open K^+^ channels and cause hyperpolarization with subsequent inhibition of L-type Ca^2+^ channels. In addition they have a nitrate-like influence and release NO via cytochrome P450 of the smooth endoplasmic reticulum (SER).
4. Sodium channel blockers (SCB) such as ranolazine inhibit the late phase of the inward sarcolemmal sodium channel and thereby prevent intracellular Ca^2+^ overload.

Ongoing clinical trials in this research area are ILIAS ANOCA [Netherlands Trial Register (Trial NL9474)], EXAMINE-CAD (EudraCT-No: 2020-004717-12), iCorMicA (NCT04674449), and WARRIOR (NCT03417388). All these studies use common antianginal medications. However, from our experience, 30%–40% of patients with angina and non-obstructive coronary arteries show refractory angina despite the use of the common antianginal drugs (20, 21). In those cases drug repurposing might play a crucial role in finding new antianginal therapy approaches.

For this review we screened the literature for possible candidates of drug repurposing in ANOCA treatment and identified two promising targets: Soluble Guanylate Cyclase (sGC) Stimulators and Endothelin-1-receptor (ET-1) blockers, both originally approved for the treatment of pulmonary hypertension (PH). Based on the molecular mechanisms of these drugs as shown in Figure 1, we believe that it is worthwhile testing them for refractory angina in clinical trials. Only then can their effectivness in the overall system of ANOCA patients be determined. We also assume that these drugs are promising only on funtional forms of ANOCA, namely an increased tendency of the coronary arteries to vasoconstrict. However, structural and functional changes in the coronary arteries can occur together or even be mutually dependent (22). Therefore, our proposed drugs may possibly influence both mechanisms (funtional and structural). Conversely, it is unlikely that primary vasodilation disorders (in the form of reduced coronary flow reserve or increased microvascular resistance without microvascular/epicardial coronary spasm) can be successfully treated with sGC stimulators or ET-1 blockers.

#### Soluble guanylate cyclase stimulators

Cyclic guanosine-3’-5’-monophosphate (cGMP) modulators have emerged as one of the most promising compounds in recent cardiovascular drug discovery (23). Genome-wide association studies have identified single nucleotide polymorphisms in genes encoding multiple components of the cGMP signalling pathway to be correlated with cardiovascular diseases (24). For instance, mutations in sGC encoding genes, leading to decreased sGC activity, have been associated with myocardial infarction risk (25). sGC is an enzyme with high affinity for NO (26) which is synthesized by numerous nitric oxide synthases (endothelial nitric oxide synthase (eNOS), neuronal nitric oxide synthase (nNOS), and the inducible isoform (iNOS)). In endothelial cells, NO is produced by eNOS and diffuses promptly to deeper located smooth muscle cells. There it stimulates sGC. This causes an intracellular increase of cGMP, triggering an assortment of reactions, e.g., vasodilation and increase of blood flow, inhibition of smooth muscle cell proliferation, leukocyte recruitment, and platelet aggregation. This axis is called the NO-sGC-cGMP pathway (27, 28). There are two different forms of sGC: the natural heme containing and the heme-free form. The heme containing form is the endogenous receptor for NO. Oxidative stress can cause the formation of reactive oxygen species (ROS). ROS on the other hand can oxidize the heme iron of sGC. This causes a loss of the heme group in the enzyme (29), which in turn leaves a dysfunctional enzyme that no longer responds to NO and is degraded by ubiquitinylation (29–31). In the late 1990s two classes of substances have been discovered that can activate both forms of sGC without NO (26, 28). Those two classes are sGC stimulators and sGC activators. sGCs stimulators are heme-dependent. They synergize with endogenous NO but can also directly stimulate the enzyme without NO. sGC activators on the other hand can activate the heme-free sGC. That means that sGC activators can activate the pathological enzyme, which can no longer be activated by endogenous NO. Both enhance cGMP synthesis independently of modulating NO levels and are thus devoid of tolerance (26).

In the mid-1990s, the pharmaceutical company Bayer looked for substances that could increase NO synthesis and thus stimulate sGC in endothelial cells. Surprisingly, they discovered NO-independent sGC stimulators. After optimization of potency, pharmacokinetic properties and specificity of this new class of drugs, riociguat was developed. It was tested in hypertensive rats and showed a dose-dependent blood pressure lowering effect. This effect lasted over numerous weeks, even when the rats were nitrate-tolerant. Riociguat was then investigated in different animal models of PH (32–34) where it improved pulmonary hemodynamics and stopped (even partially reversed) right ventricular hypertrophy and muscularization of small pulmonary arteries (33). Riociguat was selected as a drug development candidate for the treatment of different forms of PH based on its profile of excellent potency, specifity, efficacy, and safety.

The Left Ventricular Systolic Dysfunction Associated With Pulmonary Hypertension Riociguat Trial (LEPHT) showed that sGC stimulators are well tolerated in patients with advanced systolic left ventricular dysfunction and secondary PH due to heart failure. In addition, they improve cardiac index, quality of life, and pulmonary as well as systemic vascular resistance at 16 weeks of treatment. These hemodynamic improvements occurred in the absence of changes in heart rate, blood pressure, and pulmonary artery pressure. It suggested likely benefits in patients with heart failure afar those with PH (35, 36).

Vericiguat is structurally and pharmacologically distinct from riociguat and has been optimized for use in chronic heart failure patients. The VICTORIA trial investigated the effect of vericiguat in patients with HFrEF in a randomized, double-blind, placebo-controlled study design over a median of 10.8 months (37). The incidence of death from cardiovascular causes or hospitalization for heart failure was lower in the vericiguat group.

The vasodilatory effect of sGC stimulator and activators makes them promising drugs for the treatment of angina pectoris. Our research group reported about a clinical case of a 77 year-old woman with refractory angina despite conventional anti-anginal treatment (38). During an Acetylcholine (ACh) provocation test microvascular and epicardial coronary spasms could be observed. The diagnosis of a coronary vasomotion disorder was established. Unfortunately, common pharmacological therapy options to achieve satisfactory symptom control were exhausted. Therefore, we tried an off-label use of riociguat in increasing doses and weekly follow-ups until the patient described a significant drop in angina and dyspnoea symptoms. Eventually, she reported to be almost angina-free with a substantial increase in quality of life. Plasma levels of riociguat and its metabolite were analysed presenting a dose-dependent increase of plasma concentrations. The patient then undertook recurrent ACh provocation testing to evaluate the anti-vasospastic result of riociguat on the coronary arteries. Under full riociguat medication, epicardial coronary artery spasm could no longer be triggered, unlike in an earlier provocation test during treatment with “classical” anti-vasospastic drugs.

With the help of genome-wide association studies (GWAS) various loci have been recognised to be linked to coronary artery disease and myocardial infarction. To find drugs appropriate for repurposing Tragante et al. (39) formed 2 pipelines incorporating public data on 49 coronary artery disease/ myocardial infarction GWAS loci, drug-gene interactions, side effects, and chemical interactions. Those finally resulted in three ideas for drug repurposing. Riociguat was one of them, corroborating our observation.

The Coronary Vasomotor Response After Riociguat Exposure (CORONARIES) trial was organized to investigate the effects of riociguat on coronary artery disease. For unknown reasons the study was withdrawn before recruitment. A new attempt to study the potential treatment effects of sGC stimulators and activators on angina symptoms in vasomotor disorders would be highly desirable.

#### Endothelin-1- receptor blockers

Endothelin-1 (ET-1) is a potent endogenous vasoconstrictor and smooth-muscle mitogen. It can add to a rise in vascular tone and vascular smooth muscle hypertrophy. Its pathogenic role in PH has been recognized (40) as patients with primary PH have elevated plasma concentrations of endothelin 1 (41). Therefore, new drugs for PH have been developed which block endothelin receptors. As an example, bosentan is an orally active non-peptide endothelin receptor antagonist which has been proven to reduce inflammatory reactions, to avoid escalation of pulmonary vessel permeability, and to prevent progress of fibrosis in animals with pulmonary inflammation (42, 43).

However, ET-1 has been associated with the pathogenesis of coronary microvascular dysfunction (CMD) as well (44, 45). Experimental models of CMD connect greater cardiac ET-1 production with endothelial dysfunction, enhanced vascular expression of rho-kinases, and reactive oxidant species such as superoxide, and augmented vasoconstriction (46). Endothelin gene expression is regulated by a common genetic locus in chromosome 6p24 (PHACTR1/EDN1) (47). The allele, rs9349379-G, is connected with an amplified risk for atherosclerotic epicardial coronary artery disease and myocardial infarction (48). This functional single-nucleotide polymorphism (SNP) is linked to greater endothelin gene expression causing significantly elevated levels of ET-1 precursors (Big ET-1) in the plasma (47). Ford et al. (49) investigated the role of ET-1 and the aforementioned SNP in the pathogenesis of CMD and showed that the G allele was associated with higher plasma serum ET-1 and that patients with this allele had an odds ratio of 2.33 regarding coronary microvascular dysfunction. They confirmed that the SNP was linked to myocardial perfusion deficiencies on stress cardiac magnetic resonance imaging (cMRI) and exercise testing. *Ex vivo* they were able to evaluate the ET-1 related vascular mechanisms by means of wire myography with endothelin A receptor (ET_A_) antagonists including zibotentan. Patients with the SNP had unaltered small vessel reactivity to ET-1 with high affinity of ET_A_ antagonists indicating that the ET_A_ receptor might not be down-regulated in those patients. Here lies the potential treatment efficiency of a selective ET_A_ receptor antagonist.

The investigators used zibotentan for repurposing succeeding neutral results in phase 3 oncology trials (it was originally developed by Astra Zeneca for prostate cancer) since it has a good chance for clinical translation needing further phase 2 studies. It is the most selective of all orally active ET_A_ receptor antagonists, which makes it especially suitable for usage in coronary spasm therapy. Individually tailored usage of zibotentan based on genotyping is being investigated in the PRIZE trial (NCT04097314) which is expected to be completed by the end of 2023. Unfortunately, the incidence of the above mentioned SNP occurs in only about 30% of the population with microvascular angina, resulting in a high screening-failure rate. Those screening failures partake in a sub-study to perhaps identify additional expected ‘responders’ to zibotentan. There is baseline genotyping for other genetic variants in the ET-1 pathway, and phenotyping patients by quantification of microvascular disease from analysis of cMRI data. Results from this sub-study will provide a genotype database that might discover novel variants for the pathogenesis of coronary microvascular spasm which might point to other ET_A_ receptor antagonist super-responders, and enabling more patients with microvascular spasms to profit from this repurposed drug (50).

Apart from the awaited results of the PRIZE trial, there are already a few published case reports demonstrating a good therapeutic response to bosentan in patients with coronary spasm (51–53).

Since the endothelium plays an important role in the regulation of vascular tone by synthesizing vasodilator prostaglandins like prostacyclin, synthetic prostacylins might be useful in ANOCA treatment as well and represent additional candidates for drug repurposing. Agents like iloprost, which is also approved for the treatment of PH would be of interest.

## Methods of drug repurposing

How can a new indication for an already existing drug be identified? In order to discover a new target for an existing drug, different approaches are available. The general methods to identify a candidate molecule include the analysis of side effects, analysis of preclinical models as well as of the drug’s efficacy in clinical trials (54). However, there are other ways, which can lead to a more detailed characterization of a drug’s targets and its interactions with it. Affinity chromatography and mass spectrometry are used to identify potential binding partners for drugs (55, 56). In the phenotypic screening method, model systems (e.g., cell lines) are treated with molecules/drugs without knowing beforehand what targets will be affected and the following disease-relevant effects are observed and interpreted (57). Furthermore, different computational approaches are widely used to identify new targets. Signature matching, a computational approach, which uses publically accessible data (Connectivity Map, Gene expression Omnibus, Array Express) (58) analyses transcriptomic, proteomic and metabolomics data in order to compare the gene expression profiles before and after a drug treatment. This approach aims to address the following questions: “Is the differential gene expression pattern associated with a disease?” and “Do dissimilar drugs share a mechanism of action?”. If so, there might be a drug-drug similarity (59, 60).

Further computational approaches include:

1. The analyses of molecular docking, where the prediction of a binding-site complementarity between ligand (drug) and a target (receptor) (61–64) is investigated.
2. Genome wide association studies (GWAS), which identify genetic variants as novel targets, which could be shared between diseases treated by drugs and disease phenotypes (65–67).
3. Pathway or network mapping, a method favourable to use when GWAS hits are not druggable. It is a pathway-based strategy that can provide either upstream or downstream information on the GWAS-associated target. The network mapping refers to constructing a drug or a disease network based on gene expression patterns, disease pathology, protein interactions or GWAS data in order to find candidates (68–70).
4. Retrospective clinical analysis, which requires accessible databases (HER, CPRD, MHRA, Eudravigilance, FAERS, WHO, VigiBase). However, this approach often faces problems, since ethical and legal obstacles limit access to these data (71–73).

**Figure 2:**
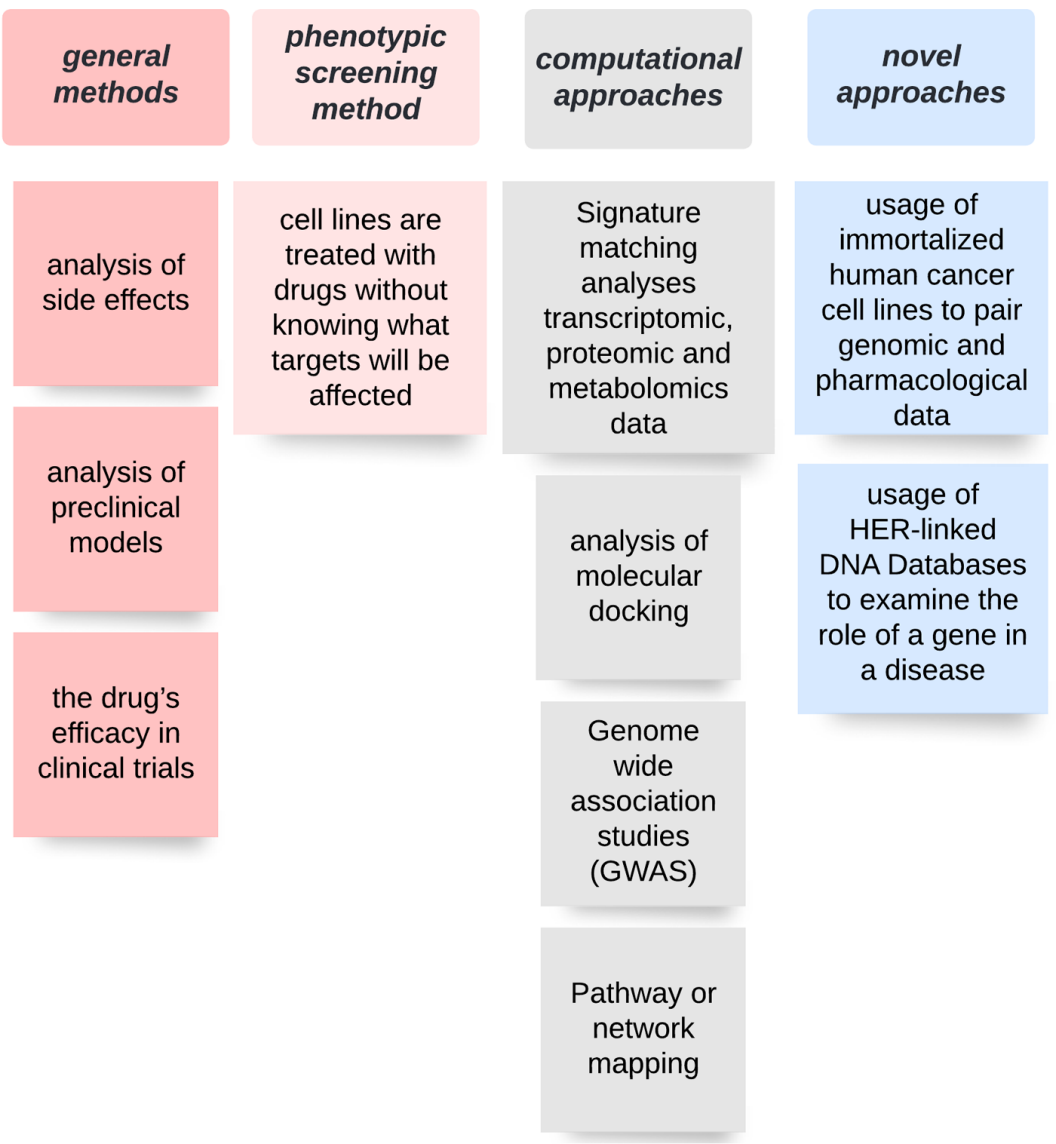
Overview of different approaches used to identify novel candidates for drug repurposing.

Novel approaches include the use of immortalized human cancer cell lines (cell viability assays) to pair genomic and pharmacological data (74) or the use of HER-linked DNA Databases (e.g., CKB) to examine the role of a gene in a disease. A good example is *PLA2G7,* a gene that was thought to be associated with vascular disease. However, since trials with darapladip, a drug blocking the gene product of *PLA2G7* in patients with coronary artery disease failed, it is now hypothesized either that gene variants are not associated with the disease or that the effect of the gene is upstream or pre-translational. In this case, the method could prove a lack of efficacy; however, the finding could also be used to identify new drug repositioning options (54, 75–77). Further approaches include the use of new sequencing technologies including large genomic data collections (78, 79) as well as the use of the world-wide-web where patients self-report data for acceleration of clinical activity and effectiveness (54, 80). Figure 2 provides an overview on different methods to identify candidates for drug repurposing.

## Conclusion

*De novo* drug development is mandatory in the ever evolving field of medical therapy. However, it is time consuming, costly and risk-prone. Drug repurposing offers faster and cheaper ways for bringing drugs for new indications to the market. Because cardiovascular disease has a high prevalence and, as a systemic disease, has linkage points with a number of other systemic diseases (e.g., diseases involving chronic inflammation, cancers), drug repurposing in cardiology appears particularly promising. Treatment of patients with coronary vasomotion disorders like in ANOCA remains challenging due to a lack of evidence-based therapy guidelines and a high proportion of patients showing refractory angina despite the use of common antianginal drugs like CCBs, beta-blockers and others. Considering the mechanisms of action two drug candidates already approved for the treatment of PH may be promising for drug repurposing in ANOCA treatment: sGC stimulators/activators and ET-1 receptor blockers. Clinical trials assessing the safety and efficacy of those candidates in the context of refractory angina due to ANOCA are needed.
